# Dietary and Endogenous Sphingolipid Metabolism in Chronic Inflammation

**DOI:** 10.3390/nu9111180

**Published:** 2017-10-28

**Authors:** Gregory H. Norris, Christopher N. Blesso

**Affiliations:** Department of Nutritional Sciences, University of Connecticut, Storrs, CT 06269, USA; gregory.norris@uconn.edu

**Keywords:** sphingomyelin, sphingolipids, ceramide, sphingosine, inflammation, obesity, atherosclerosis, diabetes, macrophage

## Abstract

Chronic inflammation is a common underlying factor in many major metabolic diseases afflicting Western societies. Sphingolipid metabolism is pivotal in the regulation of inflammatory signaling pathways. The regulation of sphingolipid metabolism is in turn influenced by inflammatory pathways. In this review, we provide an overview of sphingolipid metabolism in mammalian cells, including a description of sphingolipid structure, biosynthesis, turnover, and role in inflammatory signaling. Sphingolipid metabolites play distinct and complex roles in inflammatory signaling and will be discussed. We also review studies examining dietary sphingolipids and inflammation, derived from in vitro and rodent models, as well as human clinical trials. Dietary sphingolipids appear to influence inflammation-related chronic diseases through inhibiting intestinal lipid absorption, altering gut microbiota, activation of anti-inflammatory nuclear receptors, and neutralizing responses to inflammatory stimuli. The anti-inflammatory effects observed with consuming dietary sphingolipids are in contrast to the observation that most cellular sphingolipids play roles in augmenting inflammatory signaling. The relationship between dietary sphingolipids and low-grade chronic inflammation in metabolic disorders is complex and appears to depend on sphingolipid structure, digestion, and metabolic state of the organism. Further research is necessary to confirm the reported anti-inflammatory effects of dietary sphingolipids and delineate their impacts on endogenous sphingolipid metabolism.

## 1. Introduction

Chronic inflammation contributes to the development of many major metabolic diseases common in Western societies, including diabetes, non-alcoholic fatty liver disease, and atherosclerosis. Systemic inflammation can occur as a consequence of localized inflammatory responses in various tissues, including adipose, liver, and the distal intestine. High fat diets (HFD) have been reported to promote intestinal inflammation, gut barrier permeability, and the absorption of lipopolysaccharide (LPS) [1,2,3], which have been shown to precede increases in systemic inflammation in animal models [4]. In parallel, the accumulation of toxic lipid species, including ceramide, can drive insulin resistance, inflammation, and cell death through lipotoxicity [5]. LPS and saturated fatty acids can activate Toll-like receptor 4 (TLR4) signaling pathways to drive systemic inflammation and metabolic disease [6]. Sphingolipid metabolism plays a key role in the regulation of inflammatory signaling pathways [7]. Dietary sphingolipids show potential to influence inflammation-related chronic diseases through inhibiting intestinal lipid absorption [8,9], altering gut microbiota [10,11], activation of anti-inflammatory nuclear receptors [12,13], and neutralizing LPS [14,15]. This review will summarize sphingolipid metabolism in mammalian cells and provide an overview of the regulation of sphingolipid metabolism by inflammatory pathways. Furthermore, we will discuss the role of endogenous and dietary sources of sphingolipids in chronic inflammation and metabolic disorders, by reviewing data obtained from in vitro studies, rodent models, as well as human clinical trials. 

## 2. Sphingolipid Metabolism within Mammalian Cells

### 2.1. Sphingolipid Structure 

Sphingolipids are characterized by their amino alcohol backbone. Sphingolipids are a structurally diverse class of lipids containing >4000 distinct species with >60 possible sphingoid backbones [16]. In mammalian cells, sphingosine ((2S,3R,4E)-2-aminooctadec-4-ene-1,3-diol) is the most abundant of these backbones [17]. However, there are naturally occurring variations of chain lengths, levels of saturation [17,18], and number of hydroxyl groups [19]. For example, sphinganine ((2S,3R)-2-aminooctadecane-1,3-diol) is fully saturated, while phytosphingosine ((2S,3S,4R)-2-aminooctadecane-1,3,4-triol) is fully saturated and has a third hydroxyl group [17,18]. Shorthand nomenclature for these backbones include a “d” or “t” to indicate the number of hydroxyl groups followed by the acyl chain length and number of double bonds as seen in fatty acids. For instance, sphingosine can be written as d18:1 [20].

Upon addition of an acyl chain to the amine group, sphingosine, sphinganine, and phytosphingosine become ceramide, dihydroceramide, and phytoceramide, respectively [21]. Ceramides are the main structural backbone for the more complex sphingolipids [22]. The amide-linked acyl chain of mammalian ceramide ranges in length from 14 to 36 carbons [23,24]. However, C16-, C18-, and C24-ceramides are the most common in mammalian tissue [25]. Shorthand nomenclature for ceramide includes their acyl chain in addition to the sphingoid base, for example, C16-ceramide would be written as d18:1/16:0.

The distinguishing feature of complex sphingolipids is the head group, which replaces the hydroxyl group on the 1-position carbon. A phosphorylation at this position will produce ceramide-1-phosphate (C1P) [21]. Other additions can produce more complex sphingolipids which can be categorized either as phosphosphingolipids or glycosphingolipids. Phosphosphingolipids contain a phosphodiester linkage to their head group while glycosphingolipids have β-glycosidically linked sugars. The addition of a phosphorylcholine head group generates sphingomyelin (SM) [26]. Glycosphingolipids are characterized by their head groups, consisting of a mono-, di-, or oligosaccharide chain. Cerebrosides contain a mono- or disaccharide, which is β-glycosidically linked to the 1-position carbon. For example, glucosylceramide (GluCer) and lactosylceramide (LacCer) contain a glucose and a lactose moiety, respectively [27]. Gangliosides have longer and more complex sugar chains, which vary greatly in size and branching. Gangliosides can be classified into three groups: a-, b-, and c-series, indicating the branch point for the oligosaccharide [28].

### 2.2. Sphingolipid Biosynthetic Pathways and Subcellular Location 

**Figure 1:**
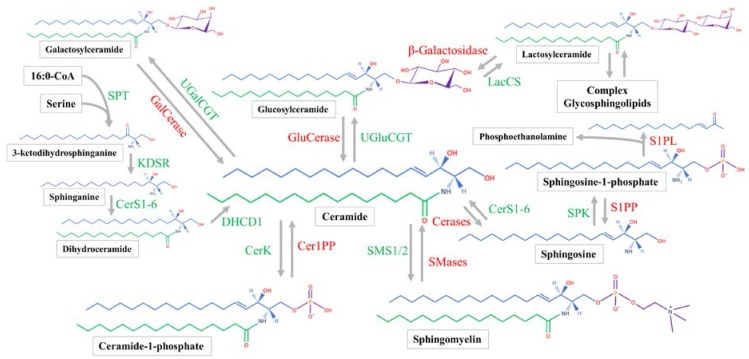
Sphingolipid metabolic pathways and enzymes described in mammalian cells. Serine and palmityl-CoA will be utilized to synthesize 3-ketodihydrosphinganine generating the sphingoid backbone (blue). The subsequent dehydrogenation and acylation will produce dihydroceramide which contains a fatty amide (green). Dihydroceramide can be desaturated to produce ceramide. Ceramide can either be used for catabolism to generate sphingosine and sphingosine-1-phosphate or one of many complex sphingolipids with an additional head group (purple). All synthesis reactions producing complex sphingolipids are reversible, while the sphingosine-1-phosphate catabolic reaction is not. Abbreviations: Cer1PP, ceramide-1-phosphate phosphatase; Cerases, acid-, alkaline-, and neutral-ceramidase; CerK, ceramide kinase; CerS1-6, ceramide synthase 1-6; DHCD1, dihydroceramide desaturase 1; GalCerase, galactosylceramidase; GluCerase, glucosylceramidase; KDSR, ketodihydrosphingosine reductase; LacCS, lactosylceramide synthase; S1PL: sphingosine-1-phosphate lyase; S1PP: sphingosine-1-phosphate phosphatase; SMases, acid-, alkaline-, and neutral-sphingomyelinase; SMS1/2, sphingomyelin synthase 1/2; SPK, sphingosine kinase; SPT, serine palmitoyltransferase; UGalCGT, UDP-galactose-ceramide galactosyltransferase; UGluCGT, UDP-glucose-ceramide glucosyltransferase.

Sphingosine synthesis is initiated by the serine palmitoyltransferase (SPT) complex utilizing palmitoyl-CoA and serine to form 3-ketodihydrosphinganine (Figure 1) [29]. 3-ketodihydrosphinganine is then reduced by 3-ketodihydrosphingosine reductase to produce sphinganine [21]. Next, one of six ceramide synthases (CerS1-6) will acylate the sphinganine to produce dihydroceramide. Each of the CerS has preferred acyl-CoA substrates [30]. After being formed by the CerS family, dihydroceramide can be converted by a desaturase enzyme to form ceramide [31]. All steps of ceramide synthesis take place on the cytosolic leaflet of the endoplasmic reticulum (ER) [32,33]. Sphingosine can only be produced by the hydrolysis of ceramide, which will be discussed later. Sphingosine phosphorylation by sphingosine kinase (SPK) will produce sphingosine-1-phosphate (S1P), which can occur at both the plasma membrane and cytosol [7].

Ceramide is the hub of sphingolipid metabolism. Before ceramide is utilized in the synthesis of complex sphingolipids, it is transported to the Golgi apparatus [34]. Mammalian cells achieve this action mainly with ceramide transfer protein (CERT) [35,36], while vesicular transport plays a minor role [37]. Once within the Golgi, ceramide is subject to several biosynthetic enzymes. Classically, the SM synthase 1/2 (SMS1/2) enzymes are located within the luminal membrane of the Golgi [38] and transfer phosphorylcholine from phosphatidylcholine to ceramide, producing diacylglycerol and SM [39]. Ceramide can also be phosphorylated by ceramide kinase to form C1P [35].

Similar to the phosphosphingolipids, glycosphingolipids are made within the Golgi from ceramide. Galactosylceramide (GalCer) and GluCer are both produced by enzymatically transferring the monosaccharides from UDP-galactose and UDP-glucose to ceramide, respectively [21]. Lactosylceramide is then produced from LacCer synthase transferring galactose from UDP-galactose to GluCer [40]. The more complex glycosphingolipids all use LacCer as a common backbone. Various enzymes can produce multiple glycosphingolipid species [41]. In general, the synthesis of complex glycosphingolipids involves the stepwise addition of sugar monomers which branch to form complex chains [28]. Complex sphingolipids are transported from the Golgi to their target location, which is typically the plasma membrane. Sphingomyelin and glycosphingolipids have been shown to be transported in a vesicle-dependent manner [42,43].

### 2.3. Sphingolipid Catabolic Pathways and Location

Sphingomyelin hydrolysis to phosphocholine and ceramide is catalyzed by alkaline, neutral, and acid sphingomyelinases (SMases) [44,45,46]. Alkaline SMase is found mainly on the plasma membrane and endosomes of the enterocytes as well as in human bile and works optimally at a pH of 8.5–9 [47,48]. Mammals have four isoforms of neutral SMase (N-SMase), which function optimally at a pH of 7.5 [49]. They are uniquely localized to the ER and nucleus (N-SMase 1), Golgi and plasma membrane (N-SMase 2), Golgi and ER (N-SMase 3), and the mitochondria (mitochondrial-associated N-SMase) [49,50]. Acid SMase is known to be the lysosomal SMase and functions at a pH below 5 [51]. Acid SMase can also be secreted from macrophages [52], and acute inflammatory stimuli will increase serum SMase activity [53]. 

Once SM is hydrolyzed, ceramide is subsequently broken down by alkaline, neutral, and acid ceramidases. All three hydrolyze the amide linkage to release a fatty acid and sphingosine. Acid ceramidase is located in the lysosome [54]. Neutral ceramidase is found in caveolin-rich plasma membrane [55,56] and can be secreted [57]. Alkaline ceramidase is in the Golgi and ER, and prefers very-long chain ceramides [58,59]. Sphingosine-1-phosphate has two possible metabolic fates. Sphingosine-1-phosphate lyase will produce phosphoethanolamine and hexadecenal [60]. The hexadecenal will be used for acyl-CoA synthesis [61]. Sphingosine-1-phosphate phosphatase (SPP1/2) can also remove the phosphate group from S1P, recovering sphingosine [62].

## 3. Sphingolipid Signaling in Chronic Inflammation 

### 3.1. Sphingolipids in Chronic Disease

Sphingolipid metabolites are well-known to play a crucial role in inflammatory signaling [7]. Cardiometabolic diseases, including atherosclerosis, type 2 diabetes mellitus (T2DM), obesity, and non-alcoholic fatty liver disease (NAFLD), share chronic inflammation as a common trait. Chronic inflammation in these disease states is characterized by a low, but consistent activation of the immune system; a consequence of the body’s failure to resolve the inflammatory response to various stimuli. Due to the low intensity of chronic low-grade inflammation, it often lacks some of the obvious clinical signs of acute inflammation, such as redness, heat, and swelling. Nevertheless, chronic low-grade inflammation promotes metabolic abnormalities, such as insulin resistance and dyslipidemia, which contribute to these metabolic diseases [63]. Many of these disease symptoms are linked to inflammation and sphingolipid metabolism. Obese patients with T2DM have elevated serum ceramide which correlates with tumor necrosis factor-α (TNF-α) concentration [64]. Weight loss in patients with non-alcoholic steatohepatitis (NASH) was shown to reduce hepatic mRNA related to ceramide synthesis [65]. Ceramide, dihydroceramide, GluCer, LacCer, SM, and S1P are all elevated in human atherosclerotic plaques [66]. All of these sphingolipids were shown to induce inflammation in human coronary artery smooth muscle cells [66]. In fact, pharmacologically inhibiting SPT by using myriocin, thus blocking sphingolipid de novo synthesis, prevented atherosclerosis progression in apolipoprotein E-knockout mice [67]. Sphingolipid de novo synthesis promotes inflammation of adipocytes as well; inhibiting SPT by myriocin or through siRNA silencing drastically reduced interleukin-6 (IL-6) and monocyte chemoattractant protein-1 (MCP-1) secretion from murine 3T3-L1 cells [68]. Ceramide, C1P, and S1P are especially important for the signaling of inflammatory pathways [69]. Each of these sphingolipids plays a distinct and complex role in cell signaling and will be discussed below.

### 3.2. Sphingomyelin and Ceramide Balance in Inflammatory Signaling

Sphingomyelin hydrolysis typically propagates inflammatory signaling. Pharmacological inhibition of acid SMase reduced TLR4 association with lipid rafts, as ceramide is needed for this complex [70]. Acid SMase is implicated in the progression of steatohepatitis [71] and hepatic reactive oxygen species generation [72]. Mice deficient in acid SMase showed resistance to hepatic steatosis and ER stress induced by HFD or methionine-choline deficient diets [73]. Oral treatment of mice with the acid SMase inhibitor SMA-7, a difluoromethylene analogue of SM, was shown to improve dextran sodium sulfate (DSS)-induced colitis and reduce cytokine production [74]. Desipramine, another inhibitor of acid SMase, blocked TNF-α-dependent production of inflammatory prostaglandins in L929 murine fibroblasts [75]. Desipramine also caused a reduction in sphingosine and S1P concentrations. When exogenous sphingosine and S1P (3 μM) were added to fibroblasts, they reversed the desipramine-related inhibition of TNF-α-dependent prostaglandin production. 

Classically, ceramide is a pro-apoptotic molecule through both caspase-dependent [76] and -independent mechanisms [77]. In most cells, endogenously produced C16-ceramide is thought to be responsible for ceramides role in programmed cell death [22,78,79,80]. Exogenous cell permeable short-chain ceramide treatments have also been shown to induce apoptosis as well [81]. As reviewed above, there are 6 isoforms of CerS that produce ceramides with differing acyl chains. These CerS isoforms seemingly have different functions in inflammation. Reducing C16-ceramide by inhibiting CerS6 protects from TNF-α-induced apoptosis, while silencing CerS1-5 and thus other ceramides species had little effect [79]. While non-inflammatory, ceramide-induced apoptosis is responsible for β-cell [82] and hepatocyte [83] death in the pathogenesis of both T2DM and NAFLD. Ceramide-induced apoptosis is related to its interactions with several downstream targets, such as ceramide-activated protein phosphatase, protein kinase B/C, and cathepsin D (for a more in-depth review see ref. [84]). 

Beyond a role in apoptosis, cellular ceramide content has been linked to inflammation and its effects on metabolic disease. Pharmacological inhibition of ceramide synthesis improves insulin sensitivity in several tissues [85]. Ceramide is a putative ligand for TLR4, to initiate pro- inflammatory pathways [86]. Ceramide also exaggerates nucleotide-binding domain, leucine-rich-containing family, pyrin domain containing 3 (NLRP3) inflammasome activation and is needed for IL-1β and IL-18 production in bone marrow-derived macrophages [87]. Ceramide-loaded low-density lipoprotein (LDL) was shown to promote IL-6 and TNF-α secretion from RAW 264.7 macrophages in a manner independent of TLR signaling [88]. Furthermore, when ceramide-loaded LDL was infused into mice for 3 h, animals became insulin resistant and had increased expression of pro-inflammatory gene expression in skeletal muscle [88]. Ceramide contributes as a signaling molecule during lipotoxicity, which plays a role in insulin resistance and development of diabetes [5]. Ceramide Synthase 6 is responsible for the production of C14- and C16-ceramide species. In obese adults, C16-ceramide content of adipose tissue is elevated compared with lean subjects, while CerS6 expression was shown to correlate with body mass index [89]. Furthermore, when CerS6 function was lost in mice, diet-induced insulin resistance and adipose inflammation were prevented [89]. Ceramide also promotes IL-1β generation in hepatocytes, as cell permeable C2-ceramide was shown to increase IL-1β production in primary rat hepatocytes [90]. Cellular ceramide modifies the production of pro-inflammatory cytokines through LPS-stimulation. J774 macrophages treated with LPS had increased cellular ceramide levels after 10 min (C18-, C18:1-, C20-, and C22-ceramides) [14]. Sakata et al. [74,91] found that inhibiting acid SMase activity using SMA-7 reduced cellular ceramide concentrations by more than 50% and inhibited the production of pro-inflammatory cytokines from LPS-stimulated cells. TLR4 association with lipid rafts is dependent on acid SMase and ceramide, as blocking acid SMase with imipramine reduced the association in differentiated THP-1 cells, and could be recovered by treatment with cell permeable C2-ceramide [70]. In summary, there is substantial evidence for endogenous ceramide’s role in cardiometabolic disease through both apoptotic and inflammatory signaling.

In contrast, there is also evidence that increasing the ceramide content of cells can prevent LPS-stimulated inflammatory responses. Inhibition and gene silencing of acid- and neutral-SMases, resulted in lower ceramide levels in LPS-stimulated macrophages and increased the secretion of TNF-α and macrophage inflammatory protein-2 (MIP-2) [14]. Furthermore, production of TNF-α decreased when ceramide hydrolysis was blocked, supporting the notion that, in this study, elevated ceramide levels in macrophages were associated with a decreased response to LPS. Macrophages derived from acid SMase-deficient mice had an increased TNF-α response to LPS-induced inflammation, while adding exogenous SMase reversed this effect [92]. Elevated serum TNF-α and reduced survival in response to LPS challenge was observed in mice lacking CerS2, which cannot produce very-long-chain ceramides [93]. These studies highlighted suggest there may be an anti-inflammatory role for ceramide in some situations. 

Sphingomyelin content can also affect inflammatory signaling. Blocking SM synthase activity will lead to a decrease in cellular SM content [94]. Macrophages derived from SMS2-knockout mice were less responsive to LPS and had reduced NF-κB activation [95]. These macrophages had decreased SM content in their cells and have reduced recruitment of TLR4 and TNF receptor to the cellular membrane. Whole body SMS2 deficiency in mice was protective against DSS-induced colitis [96]. In macrophages, SMS1 is the major isoform and its loss inhibits NF-κB activation [97]. Pharmacological inhibition of SM synthase also inhibited the inflammatory response of macrophages to LPS [98]. Additionally, hepatic overexpression SMS1 increased inflammatory markers in atherosclerotic plaques [99] and has been shown to exacerbate murine models of atherosclerosis [100]. The production of ceramide from both SM hydrolysis and the de novo pathway are important for the propagation of inflammatory signaling. However, in some instances, ceramide production is anti-inflammatory and SM synthases are necessary for inflammatory signaling. Therefore, the roles of endogenous SM and ceramide in inflammation are complex. Whether ceramide potentiates or attenuates inflammatory signaling appears to depend on the stimulus of ceramide production as well as the ceramide species generated. Basal levels of SM may be necessary for inflammatory signaling as shown by the SM synthase-knockout models being resistant to inflammatory stimuli. 

### 3.3. Role of Phosphorylated Sphingolipid Metabolites in Inflammation

Ceramide-1-phosphate involvement in inflammation signaling is not well established. However, C1P is known to be involved in the activation of cytosolic phospholipase A2 (cPLA2) in macrophages and lung cancer cells [101,102]. Consequently, C1P and ceramide kinase have been implicated in the production of eicosanoids [101,102,103]. Fatty acids on the sn-2 position of glycerophospholipids are the preferred target of cPLA2. Due to glycerophospholipid biosynthesis, these fatty acids tend to be longer and unsaturated, notably arachidonic acid [104]. Eicosanoid products from arachidonic acid metabolism are part of the propagation of obesity-related inflammation [105]. Interestingly, C1P and ceramide kinase were shown to reduce TNF-α production by inhibiting its post-translational modification by TNF-α converting enzyme (TACE) [14,106]. More research is needed to clearly define the role of C1P in inflammatory signaling. 

Traditionally, S1P has been a promoter of chemotaxis for lymphocytes [107] and a pro-survival signal [108]. As there is little S1P within tissues, the bulk is found in circulation [109]. Apolipoprotein M (ApoM) is a carrier for S1P located on HDL-particles. HDL-particles are responsible for carrying ~33% of blood S1P, with the rest bound by other lipoproteins and albumin [110]. Plasma S1P is elevated in overweight humans, as well as high-fat fed and Ob/Ob mice [109]. Antagonism of S1P receptor type 1 (S1PR1) blocked macrophage migration in response to HDL [111], which is required for the regression of atherosclerotic plaques. An agonist of the S1P receptor, FTY720, was shown to reduce atherosclerosis in mice, through lowering monocyte infiltration to artery walls and inflammation [112,113]. Treating LPS-stimulated primary macrophages with S1P inhibited the development of a pro-inflammatory M1 phenotype [114]. Conversely, intracellular sphingosine kinase activity, and therefore S1P, is required in NF-κB activation by TNF-α. Blocking sphingosine kinase results in inactivation of TNF-receptor associated factor (TAF), which is essential for TNF-α signal transduction [115,116]. Furthermore, administration of a sphingosine kinase inhibitor to Zucker lean rats protected adipose tissue against pro-inflammatory responses to LPS [117]. Conversely, when unbound S1P was added to human pulmonary epithelial cells there was an increase in NF-κB activation and ICAM-1 [118]. S1P receptors are G protein-coupled receptors and thus have multiple functions [119]. It is important to note that S1P can be a ligand for other receptors, such as peroxisome proliferator-activated receptor γ (PPARγ) [13], which is anti-inflammatory, but also lipogenic. Mice deficient in S1P lyase and thus, with elevated plasma S1P, have increased serum and hepatic lipids [120]. Furthermore, deleting sphingosine kinase in mice protected them from hepatic steatosis [121]. In the context of NASH, inhibiting S1P signaling improved the fibrotic phenotype, serum liver enzymes, and reduced macrophage recruitment without changing the sphingolipid profile [122]. Overall, some studies suggest that S1P signaling contributes to ectopic lipid deposition and hepatic inflammation, while some show inhibitory effects against inflammation. Therefore, the cellular effects of S1P in the context of inflammation are complicated.

## 4. Dietary Sphingolipids and Inflammation

### 4.1. Sphingolipids in the Human Diet

Early studies on dietary sphingolipids focused on their potential role in colorectal cancer prevention (for an in-depth review, see Ref [123]). Soon thereafter, dietary sphingolipids were recognized for their ability to impede both in vitro [124,125] and in vivo [8,9,125,126] intestinal lipid uptake/absorption. This observation has since led to studies on the effects of dietary sphingolipids on rodent [10,126,127,128,129,130,131] and human lipid homeostasis [132,133,134]. We have recently reviewed the impact of dietary sphingolipids on dyslipidemia and non-alcoholic fatty liver disease [135]. While the effects are more pronounced in animal models, promising results have been shown in human studies [135]. Most recently, the research has evolved to examine the potential inhibitory effects of dietary sphingolipids on inflammatory signaling. 

Western diets are estimated to contain 200–400 mg/day of sphingolipids [136]. In a Western diet, most dietary sphingolipids come from animal sources [136]; therefore, most dietary sphingolipids are in the form of SM [137]. In non-Western meals, SM and cerebrosides are the major sphingolipids consumed [138]. The sphingolipid content of common foods is listed in Table 1. Sphingomyelin is a major phospholipid in human milk, and infants are estimated to consume up to 150 mg of SM per day [139]. Polar lipids, like SM, are important components of milk fat globule membranes (MFGM). Milk SM feeding of rat pups has been shown to improve markers of gut maturation [140], suggesting dietary SM is important for newborn gut development. Rich sources of bovine milk SM in the human diet include cottage cheese (139 mg SM/100 g food) and buttermilk quark (74 mg SM/100 g food) [141]. The SM content of whole milk is approximately two-fold greater than fat-free milk [141]. Milk SM may be enriched in some dairy by-products due to processing. Butter serum (379 mg SM/ 100 g food) is an inexpensive dairy processing by-product which consists of the aqueous phase removed during anhydrous milk fat production [141]. Sphingomyelin is also abundant in egg yolk, as an important component of lipoproteins [136]. The SM to cholesterol ratio in egg yolk is approximately 1:2–1:4 [142,143]. 

Complex sphingolipids need to be digested before they can be absorbed. Hydrolysis begins with the removal of the head group of SM by alkaline SMase to yield ceramide and phosphorylcholine [44,47]. Alkaline SMase is bile salt-dependent, while other dietary lipids inhibit the activity of alkaline SMase [149]. Consequently, alkaline SMase activity peaks around middle of the intestines after most other lipids have been absorbed [47]. Likely due to the delay in alkaline SMase activity, some dietary SM reaches the distal intestine intact [150,151]. Glycosphingolipid digestion is less well understood. However, it has been suggested that glycosphingolipids are absorbed intact, because both bile and luminal contents failed to hydrolyze glycosphingolipids [152]. Plant-derived sphingoid bases and ceramides have been detected in the intestinal lumen of rats fed with maize GluCer [153], suggesting there may be some luminal hydrolysis of glycosphingolipids. Interestingly, rat intestines were later shown to selectively efflux plant-derived sphingoid bases into the intestinal lumen using a P-glycoprotein transporter [154], suggesting a preference by enterocytes for animal-derived sphingoid bases. Once ceramide is liberated from the complex sphingolipids, it can be further hydrolyzed to sphingosine and a fatty acid by neutral ceramidase [155,156]. Sphingosine is absorbed intact by diffusion through the epithelial cell membrane [157]. Recently, acyl-coA synthetases were shown to promote the uptake of sphingoid bases, such as sphingosine, into cells [158]. Once absorbed, the majority of sphingosine will enter the S1P lyase pathway where palmitate and ethanolamine will be produced, or, alternatively, sphingosine can be resynthesized into ceramide [155]. 

Dietary sources of SM vary in their structural composition. Milk-derived SM has a more varied composition of both fatty acids (C16:0–C24:0) and sphingoid backbones (d16:0–d19:0) compared to egg-derived SM [159]. In contrast, egg SM is composed of predominately palmitate (C16:0) and sphingosine (d18:1) [160]. Milk SM contains higher levels of the biologically inert dihydroSM [160]. Maize GluCer is a common plant sphingolipid used in dietary sphingolipid studies and the sphingoid backbone is typically 18-carbons and can contain a Δ8 desaturation and/or a 9-carbon methyl group [161]. Research studies have been conducted using phospholipid extracts, containing SM and other sphingolipids. However, these studies often do not characterize the structure of the sphingolipids. The fact a dairy cow’s diet can alter milk fatty acid composition provides a confounding variable that is important to consider when interpreting these studies [162], as it makes it more likely there will be batch-to-batch variation in sphingolipid structure.

### 4.2. Dietary Sphingolipids and Acute Inflammation

#### 4.2.1. Anti-Inflammatory Effects of Dietary Sphingolipids In Vitro

Several cell studies utilizing exogenous long chain dietary-derived sphingolipids have shown these lipids to be anti-inflammatory. Milk SM (8 µg/mL), ceramide (10 µM), and sphingosine (10 µM) inhibited LPS-induction of MCP-1 and TNF-α mRNA expression in RAW 264.7 macrophages; the effects of milk SM were found to be dependent on SM hydrolysis [15]. C8-ceramide (10 µg/mL) reduced LPS-stimulated bone marrow-derived mast cell production of IL-10 and IL-5, while having no effect on TNF-α, IL-13, or IL-6 production [163]. This same study reported a decrease in thioglycollate-elicited peritoneal macrophage production of IL-6, IL-12p40, and TNF-α in response to LPS when cells were treated with C8-ceramide. This difference was attributed to varying PI3K-Akt and MAP kinase phosphorylation between the two cell lines. Therefore, there is cell-type specificity in the effects of C8-ceramide on LPS-induced responses. Furthermore, J774 and thioglycollate-elicited peritoneal macrophages pretreated with either 10 µM C8-ceramide or sphingosine lowered LPS-induced TNF-α and MIP-2 secretion, with no effect on LPS-stimulated nitric oxide (NO) production [14]. This study proceeded to explore the potential for S1P synthesis to be anti-inflammatory. However, S1P synthesis was shown to be dispensable, as both ceramide and sphingosine inhibited LPS-induced TNF-α production in the presence of an SPK inhibitor. Exogenous sphingosine given to RAW 264.7 macrophages transfected with a PPARγ luciferase plasmid acted as a modest agonist of PPARγ [12]. PPARγ binding can repress NF-κB [164], giving a putative mechanism for the inhibition of inflammatory gene expression. 

Other sphingolipids classes have demonstrated anti-inflammatory effects when provided to cells exogenously. TLR4 typically requires lipid rafts for activity [165]. An ex vivo study in infant bowel epithelial cells, taken from viable infants, observed that pre-incubation with gangliosides inhibited LPS- and hypoxia-induced inflammation, as shown by reductions in nitrite release, IL-8, IL-6, IL-1β, eicosanoid, and hydrogen peroxide production [166]. Interestingly, sphingolipid-rich dairy fractions inhibited monosodium urate (MSU)-induced IL-8 mRNA and protein expression in THP-1 monocytes, suggesting polar milk lipids could inhibit inflammation not derived from LPS. In this study, this dairy fraction had no effect on cytokine production in response to LPS [167]. These studies demonstrate mechanistic evidence for exogenous sphingolipids other than SM and its derivatives to have inhibitory effects on inflammation. It is difficult to discern which sphingolipid metabolite is responsible for these effects, which is compounded by inflammation-induced changes in sphingolipid metabolism as reviewed above. The current evidence suggests the smaller catabolic products, such as sphingosine and ceramide, are the effectors, however, more research is needed to discern the specific bioactive component(s). 

Conversely, exogenous sphingolipids have been shown to be pro-inflammatory in some studies. Thioglycollate-elicited peritoneal macrophages expressed a pro-inflammatory phenotype when treated with exogenous SM [168]. These macrophages exhibited elevated iNOS mRNA when treated with 50–100 µM milk-derived SM, C16-SM, and C24-SM in the absence of LPS-stimulation [168]. Human embryonic kidney (HEK) cells transfected with TLR4 were shown to have an increase in TNF-α production in response to exogenous 1.5 µM C8- and C2-ceramide treatment [86]. It should be noted that CD14, which HEK cells lack, inhibited the inflammatory effect of C2-ceramide when added back in its soluble form. Both membrane-anchored and soluble CD14 are widely expressed across many cell types, but typically is less expressed in endothelial cells [169]. The disparity in CD14 expression may cause cell type-specific effects of exogenous sphingolipids on inflammation. Additionally, 50 μM of C2-ceramide has been shown to also induce ERK, JNK, and MAPK in macrophages [170]. In this study, C2-ceramide failed to activate NF-κB, thus, it only partially mimicked LPS. Furthermore, C2-ceramide had no effect on LPS-stimulated translocation of NF-κB. In another study, treating RAW 264.7 macrophages with 100 μM C2-ceramide stimulated JNK activity, but failed to activate ERK and NF-κB [171]. Conversely, one study found that treating RAW 264.7 macrophages with 10–50 μM of C2-ceramide inhibited both the activation of NF-κB and AP-1 by LPS, while reducing the production of nitric oxide and PGE [172]. The studies demonstrating pro-inflammatory effects of long chain sphingolipid use much high concentrations. Use of the cell permeable, short chain ceramides has inconsistent results and the biological relevance of these analogs is questionable. 

In summary, there are several studies supporting an anti-inflammatory role of exogenous long chain sphingolipids predominantly in response to LPS stimulation of immune cells. However, there appears to be evidence of pro-inflammatory effects of sphingolipids in the absence of LPS when given in higher dosages; therefore, more research is warranted to uncover the precise molecular mechanisms by which dietary sphingolipids affect inflammatory signaling.

#### 4.2.2. Animal Models

Effects found in cell studies have been recapitulated in animal models. Male BALB/c mice fed a MFGM-supplemented (12.5% *wt*/*wt*) chow diet for five weeks were resistant to an intraperitoneal (IP) LPS-challenge [173]. Mice fed MFGM had reduced gut permeability alongside reductions in serum IFNγ, IL-6, IL-10, IL-17, IL-12p70, MCP-1, and TNF-α after 24 h [173]. Park and colleagues [174] found similar results when feeding young male Sprague Dawley rats a 20% fat (*wt/wt*) diet supplemented with dairy-derived ganglioside (0.02% *wt/wt* of the diet). Six hours after an IP injection of LPS, the ganglioside-fed group had attenuations in LPS-induced increases in serum IL-1β and TNF-α. The intestinal mucosa of these mice also had increased ganglioside content and reductions in platelet activating factor, leukotriene B4, prostaglandin E2 (PGE), IL-1β, and TNFα, suggesting that dietary ganglioside protected against both systemic and intestinal LPS-induced inflammation. Following this study, Sprague Dawley rats fed the same ganglioside-supplemented diet were shown to have a heightened plasma and intestinal IL-10 response to LPS, while iNOS expression and subsequent nitrate and nitrite production were blunted in response to LPS [175]. This ganglioside-rich diet also improved gut barrier function markers, as shown by attenuating the LPS-induced loss of occludin protein from intestinal mucosa [175]. Another model of acute inflammation is IP injection of MSU to simulate gout. Compared to control diet, mice fed a diet supplemented with phospholipid- and sphingolipid-rich dairy fractions (0.8–7.0% *wt/wt* of the diet) were protected from increased cell migration into the peritoneal space induced by IP injection of MSU [167], suggesting that the dietary phospholipids reduced the recruitment of immune cells. Overall, animal studies examining the effects of sphingolipid and polar lipid mixtures on acute inflammation show a reduction in inflammatory responses.

#### 4.2.3. Clinical Trials

Although numerous pre-clinical studies show potential anti-inflammatory effects of sphingolipids, only a limited number of clinical trials explore the potential benefits of dietary sphingolipids in attenuating inflammation. Most of these studies examine post-prandial inflammation due to constraints on inducing sepsis in humans, and because the post-prandial inflammatory response is implicated in the risk for cardiometabolic disease [176]. Therefore, reducing this inflammatory response could have beneficial effects on cardiometabolic diseases. When overweight adults consumed saturated fat-rich meals supplemented with a sphingolipid-rich milk MFGM fraction, they had increased serum IL-10 and decreased soluble ICAM (sICAM) compared to isocaloric control meals [177]. IL-10 is an anti-inflammatory cytokine while sICAM is a biomarker positively associated with inflammation [178]. A pilot study comparing four isocaloric smoothies containing whipping cream or palm oil, made with and without MFGM, found modest effects on the postprandial response when MFGM was fed to obese adults [179]. The area under the curve for serum sICAM after consuming the palm oil-based smoothie was reduced with added MFGM, although there were no other significant effects on inflammatory markers. This study did not match fatty acids between the meals making detecting differences in post-prandial inflammation difficult. The limited number of human studies, along with the use of complex mixtures of phospholipids, makes it difficult to pinpoint the effects of sphingolipids on post-prandial inflammation.

### 4.3. Dietary Sphingolipids Attenuate Models of Chronic Inflammation

#### 4.3.1. Colitis Models

Dietary milk SM (0.1% *wt/wt*) was shown to modestly ameliorate DSS-induced colitis in a PPARγ-dependent manner despite elevations in immunostimulatory gene expression including Ccl11/19/20 and Cxcl9/11 [12]. The authors suggested that the simultaneous upregulation of anti-inflammatory genes may be responsible for the decrease in disease severity. Furuya et al. [180] also found dietary SM (0.1% *wt/wt*, unspecified source) to be protective against DSS-induced colitis by reducing myeloperoxidase and histological damage. BALB/c mice who were fed a maize GluCer-supplemented (0.1% *wt/wt*) diet expressed lower colonic concentrations of several inflammatory cytokines in response to 1,2-dimethylhydrazine (DMH), including TNF-α, regulated on activation, normal T cell expressed and secreted (RANTES), macrophage colony stimulating factor (M-CSF), and interferon-inducible T-cell alpha chemoattractant (I-TAC) [181]. In another study, DSS-treated BALB/c mice fed GluCer (0.1%) also had reduced colonic cytokines including IL-1α/β, sICAM1, IL-16, interferon gamma-induced protein 10 (IP-10), monokine induced by gamma interferon (MIG), and TIMP metallopeptidase inhibitor 1 (TIMP-1) [182]. These studies together suggest a reduction in inflammation and recruitment of immune cells into the colon during colitis when fed dietary SL. In contrast, Fischbeck and colleagues [183] found 4 mg/day of egg SM given via gavage exacerbated colitis and apoptosis in both DSS and IL-10^−/−^ colitis mouse models. Colitis increased ceramide content in the intestinal epithelial cells of these mice. This study may have seen pro-inflammatory effects due to the large dose of SM administered by gavage as compared to the studies showing improvements in colitis where the sphingolipid was mixed into the diets, and, thus, spreading the dose throughout the day. This could be related to the pro-inflammatory effects of higher doses of sphingolipids reported in cell studies.

#### 4.3.2. High-Fat Diet-Induced Inflammation

We have recently reported that feeding 0.25% (*wt/wt* of the diet) of milk SM to high-fat fed mice reduced circulating endotoxin activity in C57BL/6J mice fed a Western-type diet for four weeks [10]. Circulating endotoxin activity contributes to the chronic low-grade inflammation found in obesity. This change was accompanied by fecal reductions in Gram-negative bacteria and an increase in *Bifidobacterium* [10], suggesting dietary sphingolipids can modulate the gut microbiota in a potentially beneficial manner. In a follow-up study, we examined the effects of feeding 0.1% (*wt/wt*) milk SM or egg SM during an obesogenic diet [131]. In that study, both SM-fed groups reduced epididymal adipose tissue inflammation and serum CCL2 concentration. Furthermore, 0.1% milk SM was shown to be effective at reducing the concentrations of other serum cytokines (TNF-α, MIP-1β, IFNγ, and IL-6) compared to obese HFD-fed controls, although SM had little impact on colon and mesenteric adipose inflammatory markers or gut microbiota composition in this study [15]. Lecomte et al. [184] examined the effects of feeding mice HFD (40% kcal from fat) supplemented with 2% (*wt/wt*) milk polar lipids (MPL), containing ~0.3% milk SM. The mice given MPL were protected against the effects of the diet compared to the HFD control. The intestinal expression of Mucin2 (Muc2) was increased by MPL suggesting increased intestinal integrity. The adipose tissue of MPL-fed mice also had reduced CD68 mRNA, a marker of macrophage infiltration. In contrast, mice supplemented with soy polar lipids showed an increase in epididymal adipose mRNA for TNF-α, CCL2, and LPS-binding protein compared to HFD control. The reduction in inflammation may be related to MPL ability to reduce intestinal leakiness [173], thus limiting endotoxin translocation. The complex phospholipid mixture complicates the interpretation of this study. Not only have dietary phospholipids been reported to have their own beneficial health effects [185], but they also can alter milk SM-derived ceramide appearance in the lymph [186], suggesting more milk SM is being digested and absorbed. However, a major difference in the composition of MPL and soy polar lipids are the sphingolipids, suggesting they may have contributed to the reduction of inflammation in this study [184]. As opposed to the beneficial effects discussed above, feeding ox brain sphingolipids (0.1% of the diet) exacerbated atherosclerosis development in low-density lipoprotein-receptor-deficient mice fed a chow diet [187]. Overall, these chronic feeding studies suggest a potential for sphingolipids to reduce HFD-related inflammation and cardiometabolic disease, but more studies are needed to fully understand the mechanisms. Results from animal studies examining the effects of dietary sphingolipids on inflammation are summarized in Table 2.

### 4.4. Fumonisins as Inhibitors of Sphingolipid Metabolism and Impact on Inflammation

Fumonisins are a group of mycotoxins that can interfere with sphingolipid metabolism [188]. Fumonisins have been found as food contaminants associated with a number of diseases linked to the consumption of moldy corn, including esophageal cancer [189] and liver cancer [190,191] in humans. Administration of fumonisin B1 to animals causes hepatoxicity and nephrotoxicity, resulting in cancer of the kidney and liver [188]. The toxicity of fumonisins stems from their structural similarities to sphingoid bases, which enables them to act as potent inhibitors of ceramide synthase [188]. Ceramide synthase inhibition results in an increase in cellular free sphingoid bases, primarily sphinganine, since these sphingoid bases cannot be used for complex sphingolipid synthesis [188]. Thus, fumonisin-induced disease appears to be related to its effects on the de novo synthesis of complex sphingolipids as well as reacylation of sphingosine derived from complex sphingolipid turnover and/or dietary sources [188]. Fumonisin toxicity in animals has been linked with TNF-α production [192,193]. Fumonisin B1 administration in mice resulted in increases in TNF-α expression in liver [192]. Peritoneal macrophages isolated from rodents treated with fumonisin B1 had increased TNF-α secretion in response to LPS stimulation [193]. In contrast, fumonisin B1 administration was shown to reduce TNF-α concentrations in the ileum of rats subjected to splanchnic artery occlusion/reperfusion, thus exerting anti-inflammatory effects in this model [194]. Similar to exogenous sphingolipid treatments, effects of fuminosin appear to be modulated by intracellular sphingolipid balance and are cell-type specific, time- and dose-dependent.

## 5. Conclusions

**Figure 2:**
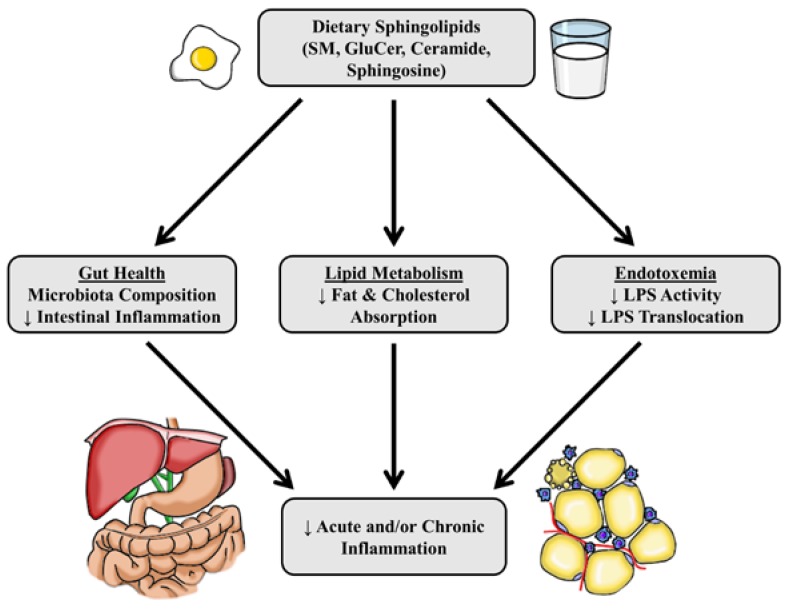
Potential effects of dietary sphingolipids relevant to acute and/or chronic inflammation. Abbreviations: GluCer, glucosylceramide; LPS, lipopolysaccharide; SM, sphingomyelin; ↑, increase; ↓ decrease.

Classically, sphingolipids are understood to be second messengers that propagate the inflammatory response. However, most studies examining sphingolipid metabolism and inflammation show their role is more complex. This idea is compounded by the evidence showing exogenous long chain sphingolipids may dampen both acute and chronic inflammatory responses in cell and animal models (Figure 2). The reported anti-inflammatory properties of dietary sphingolipids are in contrast to the observation that most cellular sphingolipids play roles in augmenting inflammatory signaling. Overall, this suggests the regulation of inflammation is impacted by the balance of sphingolipids in addition to abundance. However, more research is needed to fully elucidate the mechanisms by which sphingolipids act in inflammation, and to further test the possible beneficial clinical impacts of dietary sphingolipids on chronic inflammation-related metabolic diseases. 

## Figures and Tables

**Table 1:** Sphingolipid content of foods.

Sphingolipid	Dietary Sources	Content (mg/100 g)	Ref
Sphingomyelin	Bovine Milk, Whole	9	[142]
	Beef	44–69	
	Egg	82	
	Cottage Cheese	139	
	Mackerel	224	
	Chicken Liver	291	
Ceramide	Rice Bran	5.6	[144]
	Soybean	11.5	[145]
Cerebroside	Rice Bran	11.5	[144]
	Soybean	310	[145]
	Corn	11.5	[146]
	Soybean	20	[147]
	Amaranth Grain	39	[147]
Ganglioside	Bovine Milk, Whole	0–11	[148]
	Mackerel	6.48	
	Chicken Egg Yolk	15.9	
	Chicken Liver	29	
Total Sphingolipids	All Sources	200–400 mg consumed/day	[136]

**Table 2:** Animal studies examining the effects of dietary sphingolipids on inflammation.

Sphingolipid Source	Animal Model	Diet and Duration	Results	Ref
Sphingomyelin	Male JcI:ICR mice ( *n* = 10/group)	Mice were acclimated to AIN-76 diet for 3 days before the addition of 0.1% SM ( *wt/wt*; unspecified source). Three days following SM in diet, 2% DSS added to drinking water for 1 week	SM: ↓weight of intestine, disease activity score, intestinal MPO activity, weight loss, histological damage of the mucosa, ↑IgA in large intestine without DSS treatment	Furuya et al. 2008 [180]
Sphingomyelin	Male and female epithelial and hematopoietic cell specific PPARγ^−/−^ mice (MMTV-LTR-promoter) ( *n* = 10/group)	AIN-76A diets ± 0.1% ( *wt/wt*) milk SM (MSM) with 7 days of diet. Day 8: Injection of azoxymethane (10 mg/kg). Day 13: 2% DSS added to drinking water	PPARγ^+/+^ Mice: MSM: ↓ recovery time from DSS, ↑ mRNA of pro- and anti- inflammatory genes	Mazzei et al. 2011 [12]
			PPARγ^−/−^ Mice: MSM: ↓recovery time from DSS, ↑survival, ↑mRNA of pro- and anti- inflammatory genes	
Sphingomyelin	C57BL/6J mice ( *n* = 17/group); IL-10^−/−^ mice (*n* = 5/group)	Colitis was induced in C57BL/6J mice using 0.2% DSS in drinking water. IL-10^−/−^ develop spontaneous colitis. Mice were gavaged with either water, 4 mg, or 8 mg egg-derived SM (ESM) suspended in water	4 mg ESM C57BL/6: ↑ intestinal epithelial cell ceramide, colitis score, weight loss, epithelial damage score, apoptotic cells, cathepsin D, caspase-3 activity	Fischbeck et al. 2011 [183]
			8 mg ESM C57BL/6: ↑sphingosine in IECs with no DSS, ↑SM in IECs with DSS treatment, ↔ colitis score, weight loss, epithelial damage score, apoptosis	
			4 mg ESM IL-10^−/−^: ↑ colitis score and weight loss	
Sphingomyelin	Male C57BL/6J mice ( *n* = 10/group)	Mice were fed HFD (45% kcal from fat) ± 0.25% ( *wt/wt*) egg- or milk-derived SM (ESM or MSM) for 4 week	MSM: ↓serum LPS, ↓fecal % Gram-negative bacteria, ↑fecal % *Bifidobacterium*, ↔ FITC-dextran gut permeability	Norris et al.*.* 2016 [10]
			ESM: ↔ serum LPS	
Sphingomyelin	Male C57BL/6 mice ( *n* = 14)	Mice were fed HFD (60% kcal from fat; 0.15% cholesterol by weight) ± 0.1% ( *wt/wt*) egg- or milk-derived SM (ESM or MSM) for 10 week	MSM: ↓serum CCL2, ↓adipose inflammatory mRNA (F4/80, TNF-α)	Norris et al. 2017 [131]
			ESM: ↓serum CCL2, ↓adipose crown-like structures, ↓adipose inflammatory mRNA (F4/80, CD68, CD11c, CCL2, TNF-α), ↓ hepatic CCL2 mRNA	
Glucosylceramide	Female BALB/c mice ( *n* = 10–20/group)	Mice were acclimated to AIN-76 test diet ± GluCer (0.1% *wt/wt*) for 10 days. Drinking water was supplemented with 0.2% DSS for 15 days	GluCer: ↓weight loss, ↓colonic MPO, TIMP-1, MIG, IP-10, IL-16, IL-1β, IL-1α, sICAM-1	Arai et al. 2015 [182]
Glucosylceramide	Male BALB/c mice ( *n* = 8/group)	Mice fed AIN-76A diet ± 0.1% ( *wt/wt*) maize GluCer for 7 week with weekly IP injection of DMH	GluCer treatment: ↓aberrant crypt foci, ↓ colonic IP-10, I-TAC, MIG, RANTES, TNF-α, IL-23, M-CSF	Yamashita et al. 2017 [181]
Ganglioside	Male Sprague-Dawley rats ( *n* = 16/group)	Rats fed a 20% *wt/wt* HFD containing 0.1% ganglioside (GG) for 2 week before LPS IP injections (3 mg/kg) and samples collected 6 h later	GG: ↓ intestinal cholesterol, PAF, PGE2, LTB4, interleukin (IL)-1β, TNF-α and ↓ plasma LTB4, TNF-α, IL-1β, ↑ intestinal GG content	Park et al. 2007 [174]
Milk Fat Globule Membrane (MFGM)	Male BALB/c mice ( *n* = 6/group)	Mice fed AIN-76A or modified AIN-76A to include 12.5% MFGM for 5 week. Mice were IP injected with LPS (10 mg/kg) and gut leakiness and serum cytokines were measured at 24 and 48 h	MFGM 24 h: ↓ gut permeability to FITC-dextran ↓ IFNγ, CCL2, TNF-α, IL-3, IL-17, IL-12p70 compared to control diet at 24 h	Snow et al. 2011 [173]
			MFGM 48 h: Similar to MFGM 24 h with RANTES, IL-5, and IL-1β compared to 24 h controls, as control mice did not survive 48 h	
Mixed Phospholipids	C57BL/6J mice ( *n* = 12/group)	Mice were fed 40% kcal from fat (mostly palm oil) for 8 week on one of three diets: HFD control, HFD-soy phospholipids (SPL), HFD-milk phospholipids (MPL)	MPL: ↑ Muc2 staining in colon, ↓ adipose CD68 mRNA compared, ↓ jejunal goblet cell count SPL: ↑ epididymal adipose MCP-1, TNF-α, LBP, leptin mRNA	Lecomte et al. 2015 [184]

*Abbreviations and symbols:* CCL2, C-C motif chemokine ligand 2; DSS, dextran sulfate sodium; FITC, fluorescein isothiocyanate; GluCer, glucosylceramide; HFD, high fat diet; IECs, intestinal epithelial cells; IFNγ, interferon gamma; IgA, immunoglobulin A; IP, intraperitoneal; IP-10, interferon gamma-induced protein 10; ITAC, interferon-inducible T-cell alpha chemoattractant; LTB4, leukotriene B4; LPS, lipopolysaccharide; M-CSF, macrophage colony-stimulating factor; MFGM, milk fat globule membrane; MIG, monokine induced by gamma interferon; MPO, myeloperoxidase; PAF, platelet-activating factor; PGE2, prostaglandin E2; PPARγ, peroxisome proliferator-activated receptor gamma; RANTES, regulated on activation, normal T cell expressed and secreted; sICAM-1, soluble intercellular adhesion molecule 1; SM, sphingomyelin; TIMP-1, TIMP metallopeptidase inhibitor 1; TNF-α, tumor necrosis factor-alpha; ↑, increase; ↓ decrease; ↔ no change.
